# Methicillin-Resistant *Staphylococcus aureus*: Risk for General Infection and Endocarditis Among Athletes

**DOI:** 10.3390/antibiotics9060332

**Published:** 2020-06-18

**Authors:** Mariarita Brancaccio, Cristina Mennitti, Sonia Laneri, Adelaide Franco, Margherita G. De Biasi, Arturo Cesaro, Fabio Fimiani, Elisabetta Moscarella, Felice Gragnano, Cristina Mazzaccara, Giuseppe Limongelli, Giulia Frisso, Barbara Lombardo, Chiara Pagliuca, Roberta Colicchio, Paola Salvatore, Paolo Calabrò, Raffaela Pero, Olga Scudiero

**Affiliations:** 1 Department of Biology and Evolution of Marine Organisms, Stazione Zoologica Anton Dohrn, Villa Comunale, 80121 Naples, Italy; mariarita.brancaccio@szn.it; 2 Department of Molecular Medicine and Medical Biotechnology, University of Naples Federico II, Via S.Pansini 5, 80131 Naples, Italy; cristinamennitti@libero.it (C.M.); cristina.mazzaccara@unina.it (C.M.); giulia.frisso@unina.it (G.F.); barbara.lombardo@unina.it (B.L.); chiara.pagliuca@unina.it (C.P.); roberta.colicchio@unina.it (R.C.); paola.salvatore@unina.it (P.S.); 3 Department of Pharmacy, University of Naples Federico II, 80131 Naples, Italy; slaneri@unina.it (S.L.); ade.franco@studenti.unina.it (A.F.); margherita.debiasi@unina.it (M.G.D.B.); 4 Department of Cardio-Thoracic and Respiratory Sciences, Università degli Studi della Campania “Luigi Vanvitelli”, 80138 Napoli, Italy; arturocesaro@hotmail.it (A.C.); limongelligiuseppe@libero.it (G.L.); 5 Center of Excellence for Research on Cardiovascular Diseases Università degli Studi della Campania “Luigi Vanvitelli”, 80138 Napoli, Italy; fimianifabio@hotmail.it; 6 Department of Translational Medical Sciences, Università degli Studi della Campania “Luigi Vanvitelli”, 80138 Napoli, Italy; elisabetta.moscarella@unicampania.it (E.M.); paolo.calabro@unicampania.it (P.C.); 7 Division of Cardiology, Department of Translational Medical Sciences, Università degli Studi della Campania “Luigi Vanvitelli”, 80138 Napoli, Italy; gragnano.f@gmail.com; 8 Task Force on Microbiome Studies, University of Naples Federico II, 80100 Naples, Italy; 9 Ceinge Biotecnologie Avanzate S. C. a R. L., 80131 Naples, Italy

**Keywords:** *Staphylococcus aureus*, infections in athletes, transmission, physical contact

## Abstract

The first studies on *Staphylococcus aureus* (SA) infections in athletes were conducted in the 1980s, and examined athletes that perform in close physical contact, with particular attention to damaged or infected skin. Recent studies have used molecular epidemiology to shed light on the transmission of SA in professional athletes. These studies have shown that contact between athletes is prolonged and constant, and that these factors influence the appearance of infections caused by SA. These results support the need to use sanitary measures designed to prevent the appearance of SA infections. The factors triggering the establishment of SA within professional sports groups are the nasal colonization of SA, contact between athletes and sweating. Hence, there is a need to use the most modern molecular typing methods to evaluate the appearance of cutaneous SA disease. This review aims to summarize both the current SA infections known in athletes and the diagnostic methods employed for recognition, pointing to possible preventive strategies and the factors that can act as a springboard for the appearance of SA and subsequent transmission between athletes.

## 1. Introduction

For many years, researchers and sports doctors have been investigating the causes and rapid spread of *Staphylococcus aureus* (SA) infections among athletes [1,2,3,4]. Bacterial infections in both professional athletes and the non-sporting population are known to be caused by both Gram-positive (*Staphylococcus aureus*) [1,2,3,4,5] and Gram-negative (*Helicobacter pylori*) [6,7,8,9] bacteria, and often these infections can generate disorders of various organs, leading to serious chronic diseases and even cancer [7,8,9]. In addition, the engraftment of these pathogens and their propagation can often be caused by a dysregulation of the microbiome [5,10,11,12]. In this scenario, a key role is played by the immune system, and the cellular and peptide components that are responsible for defending the body [13,14,15,16]. SA is the most dangerous of all the numerous and common bacteria belonging to the *Staphylococcus* genus. These spherical Gram-positive bacteria frequently cause skin infections, but can also cause pneumonia, heart valve infections and bone infections [1,5]. In fact, initially, SA was considered to be a microorganism capable of causing only nosocomial infections. However, numerous studies have since shown that this pathogen causes numerous infections that spread rapidly, especially among professional athletes [1]. The ease with which SA infects human skin is due to the presence of ecological niches in the skin tissue that allow the pathogenic microorganism to survive [5].

**Figure 1:**
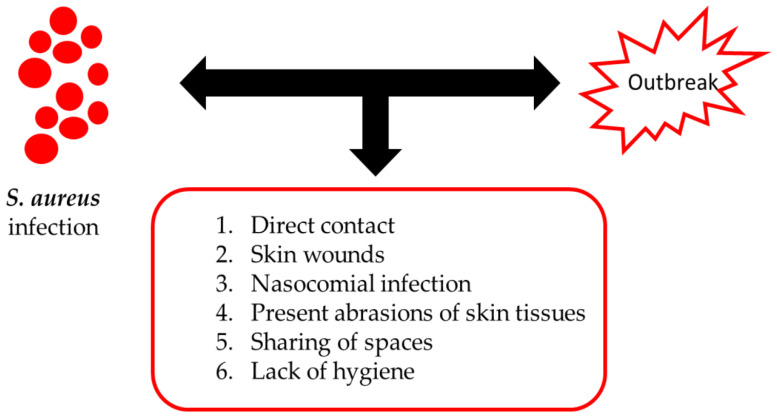
Risk factors associated with *Staphylococcus aureus* infections.

The appearance of SA epidemics causes a rapid spread of the microorganism. In particular, repeated skin-to-skin contact between athletes during sports practices is the main cause of the manifestation and spread of SA infection. Furthermore, the high incidence among athletes is also caused by the sharing of spaces or common objects, such as equipment, benches, changing rooms and razors [2,3]. The factors that contribute to the spread of the SA epidemic can be classified into three categories (Figure 1): a) direct contact (contact sport), b) rival skin wounds and c) nosocomial disorders. In addition, in athletes, the onset of this infection can be caused by other factors, such as abrasions of skin tissues, the sharing of common spaces and a lack of hygiene (Figure 1).

During these outbreaks, the infection can cause the team to stop or drop out of the league. Furthermore, outbreaks of infectious diseases can also spread within sports clubs [4,17]. The two most common sources of SA spread are contaminated hands and physical contact with athletes [2,18]. Multimodal peaks are representative of SA outbreaks, and include a risk factor for these outbreaks during physical contact sports [19]. These studies highlight how disease transmission can be caused by skin lesions that act as an entry point for infectious organisms.

The primary goal of this review is to provide a better understanding of SA infections and the potential relationship with the associated sports activity. This review summarizes both the current insights into the sports activity related to SA infections and risk factors, and the latest diagnostic methods for recognition. Our study provides novel and evaluable information on the effects of elite physical activity on SA infections, and involves different fields of applications, such as microbiology, biochemistry, internal medicine and sports medicine.

## 2. *S. aureus* outbreaks in Athletes

It is known that, during the sports season (the competitive period of a sports league), outbreaks of SA can occur [20,21]. The establishment of this situation, in the competitive period, is due to a greater opportunity for contact between athletes and therefore a greater possibility of spreading the infection. Recently, a high rate of SA infection and a methicillin-resistant SA (MRSA) epidemic was reported in men’s soccer teams during the regular season [1]. In 2003, a study was conducted on the St. Louis Rams football team during the football season [1]. During the football season, eight MRSA infections occurred among five of the 58 players (9%) belonging to the team. All infections developed at the turf abrasion sites. No MRSA was found in nasal or environmental samples; however, methicillin-sensitive SA was recovered from hot tubs, taping gels, and 35 of the 84 nasal swabs from players and staff members (42%). These data indicate that the sharing of common spaces such as whirlpools and the playing field, or taping gel, can be a vehicle for the spread of SA infection [1]. Therefore, the clinical “surveillance” of athletes participating in physical contact sports during championship events is essential for preventing and controlling the emergence of SA outbreaks.

## 3. Importance of Fomites in *S. aureus* Infection Outbreaks

**Figure 2:**
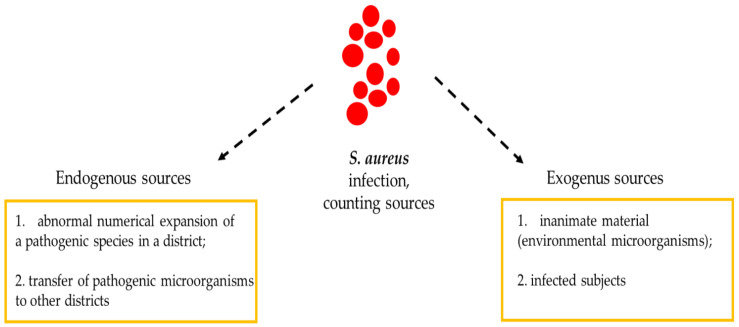
Sources of contagion for *Staphylococcus aureus* infections.

Athletes who have contracted skin infections caused by SA are more likely to have a recurrence if the fomites were contaminated with SA. The ease with which MRSA spreads among athletes is due to the different conditions faced during training and competitions. In fact, athletes can exhibit repeated skin-to-skin contact; abrasions of skin tissues that, if left uncovered, can facilitate the spread of MRSA; the sharing of spaces; a lack of hygiene, such as a lack of washing hands or taking a shower after training or post-race; and the sharing of tools and toilets [1,3,20,21,22,23]. It is true that the pathogenic microorganism is commonly spread through droplets transported from the area (aerosol), but direct contact with nasal secretions or fomites (contaminated objects) plays a fundamental role in the rapid spread of the infection. However, in some cases, it is possible that the strain responsible for the appearance and propagation of SA-induced disturbances was acquired from a non-nasal endogenous source or environmental sources (Figure 2).

In this regard, Haghverdian and collaborators [24] conducted a study on the transmissibility of MRSA within two different sports groups: a basketball team and a volleyball team. The purpose of the study was to underline how the field and the ball used could be the vehicle of the infection, or the fomite for the appearance and transmission of the pathology from SA. This study showed that SA remained on the ball for 72 hours, which is why the hygiene and disinfection of common areas are of fundamental importance, in order to reduce SA infection. In addition, Creech and colleagues [20] carried out a study on 126 subjects, and stressed that the appearance of MRSA increases during the season (4–23%). This increase is not due to the appearance and development of repeated outbreaks, and only one outbreak or one infected athlete is enough to increase the spread and the percentage of individuals infected, thanks to SA’s ability to escape from common antibiotic therapies. Another important study on the appearance and spread of SA was conducted by Jiménez-Truque and co-authors [25]. They subjected 377 athletes to a nasal swab, both at the time of enrolment within the study and every month, in order to monitor the state of health and the presence of SA or the appearance of MRSA. Of the 377 subjects, 224 practiced contact sports and 153 practiced contactless sports. Most of the athletes were male (57.3%) and Caucasian (74.3%). During the two years of study, it was seen that, in 95% of SA cases, the affected subjects practiced contact sports.

Furthermore, Mascaro and co-workers [26] conducted a cross-sectional study among contact or collision sports athletes in Italy. They found that SA was carried by 42% of 238 enrolled athletes. Colonization was associated with the sharing of sports equipment, not taking a shower immediately after training, and a previous history of pharyngitis or sinusitis and skin manifestations.

Moreover, recently Yokomor and co-authors [27] have reported, for the first time, a fatal case of a 20-year-old Japanese college athlete with community-acquired methicillin-resistant SA USA300 Clone. He had several abrasions on his extremities caused by playing rugby football. The authors strongly hypothesize that the USA300 clone of this case was derived from the nasal cavity of one of his teammates. In non-epidemic settings, close contact with a person who has a skin infection associated with SA infection has been reported to cause an outbreak of MRSA [28]. The continuous monitoring of SA strains in the environment has contributed to effective strategies that can be used to prevent SA infection. For example, athletic associations should implement dissemination and prevention programs that encourage body and hand hygiene through the use of antimicrobial soaps immediately after any training or competition practice [29,30]; apply environmental decontamination rules to common places such as changing rooms, bathrooms, and benches [28]; and discourage the use of shared objects, such as razors and towels [3,29]. As a result of active surveillance, a consensus was reached on optimal approaches for infection control among athletes [31], which could be achieved by implementing critical nostril SA screening to prevent skin and soft tissue infection.

## 4. *S. aureus* Infection in Athletes

**Figure 3:**
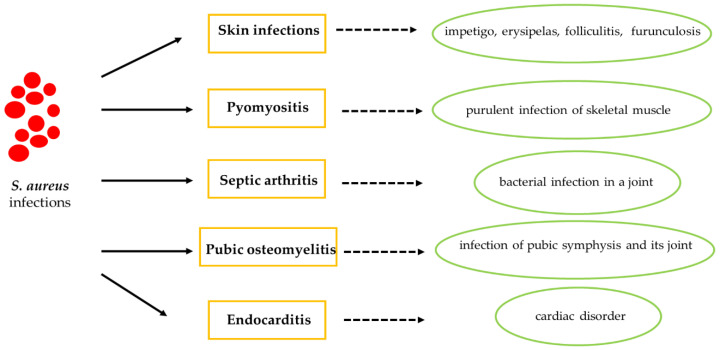
Common infections of *Staphylococcus aureus*.

SA can cause numerous disorders, such as skin infections, pyomyositis, septic arthritis, pubic osteomyelitis and endocarditis (Figure 3). The long-term nasal transport of SA is a known risk factor for the spread of skin infection in sports including physical contact [20]. However, SA can spread among athletes not only by the presence of skin infections, but also thanks to its ability to survive in the common areas of a sports facility [21,24]. In fact, SA resides in the anterior nostrils of individuals, the main reservoir for the pathogen, predisposing athletes to subsequent infections. In contrast, SA infections account for 30% of endocarditis cases in hospitalized non-athletic patients [32,33].

### 4.1. Cutaneous Tissue in Athletes with S. aureus Infection

**Figure 4:**
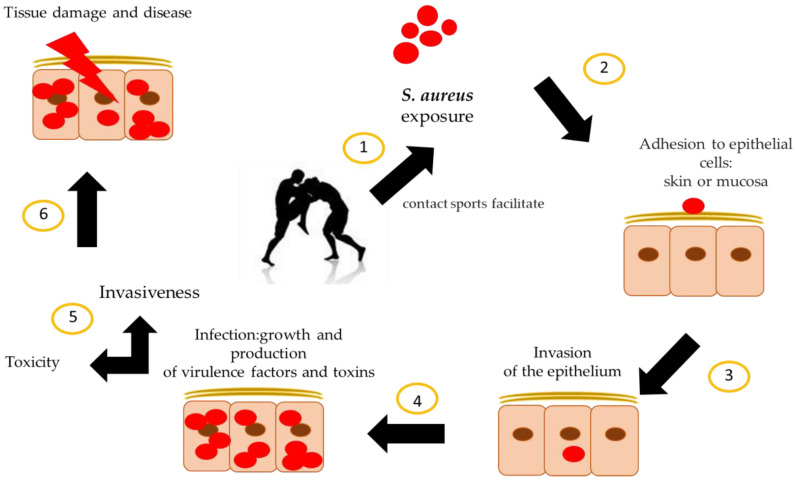
Mechanism of *Staphylococcus aureus* infection in skin tissue.

The cutaneous tissue represents the largest organ of the human body [34]. Therefore, in contact sports, there is an increased risk of contracting SA infections (Figure 4) [35,36,37]. In fact, it has emerged that skin-to-skin contact represents a central cause of transmission of SA among athletes [1]. Many studies have highlighted how the surface of dry, salty, low pH skin prevents the correct growth of SA [38]. However, an athlete’s skin, soaked in sweat, provides a microenvironment suitable for SA growth. Therefore, the release of sweat from the skin and the consequent contact between athletes are the key points of the transmission of infections caused by SA in contact sports such as football, basketball and rugby [39]. In addition, the nasal transport of SA has a strong ability to colonize skin tissue [40]. Furthermore, it has been shown that the sweat glands, the sebaceous glands and the hair follicles have a single microbiota [41]; in this case, the sebaceous glands secrete the lipid-rich sebum, and with this hydrophobic coating, they are able to protect and lubricate the hair and skin, acting as an antibacterial coating generating a molecular defense mechanism [42]. However, the relationship between exercise-induced sweating and the transmission of SA in sports by physical contact between athletes remains unclear. In this scenario, two factors may be involved in the transmission of SA in an athletic environment. First, nasal carriers also carry the pathogen on their hands. Therefore, not only are contaminated hands considered to be a likely source of transmission, but hands, in many cases, act as vectors for the transmission of nasal SA. Secondly, SA can also live on the skin, which means that it can easily spread from one person to another through sweat and subsequent contact. The latter hypothesis is considered the dominant mode of transmission. SA is more present on the skin surface due to the sweat produced during exercise in nasal carriers [28]. Although SA is found on the skin, the nose appears to be the main reservoir for its replication and transmission to other sites in the body. In fact, in the case of SA infection, the use of a topical intranasal antibiotic is suggested, which will have the task of temporarily blocking the transfer of SA from the nose to other colonization sites [43]. This is because, in most cases, nasal SA isolates are often identical to the strains that subsequently cause clinical infections [44,45]. These infections show an endogenous origin [46,47]. This knowledge allows us to affirm that direct physical contact with biological fluids is one of the main sources of the diffusion of SA [48]. Suzuki and Tagami [28] examined the SA of the skin surface before and after exercise, showing that the density of the nasal SA was related to that of the SA of the skin surface. This study indicates that sweat during exercise promotes the appearance of SA in nasal carriers. Recently, direct evidence of the existence of SA on the surface of the skin has been reported in healthy adult males after participating in high-intensity resistance exercises [49]. As a result, sweat during exercise seems to play a crucial role in the appearance and transmission of the infection. Therefore, the chances of players on the team passing SA to other team member increases during training and matches.

### 4.2. Skin Disorders

SA is the most common sports-related skin infection. SA infection may manifest as impetigo, erysipelas, folliculitis [50,51,52] and furunculosis [53]. Impetigo is characterized by erythematous, yellow-crusted, scaling plaques, and erysipelas [50,51]. Folliculitis manifests as small follicular pustules. These disorders occur in athletes who play contact sports such as rugby, judo and wrestling [51,52]. Furunculosis outbreaks, however, have also been noted in soccer and basketball athletes. Direct contact with furuncles was the main cause of transmission, followed by sharing equipment. Gym bags and wrestling mats also appear to facilitate the transmission of SA [52]. In order to reduce transmission and infection, the immediate isolation of the sick athlete is necessary [50,51,52,53]. If the incidence of infection is low, bandaging can be a reasonable means of preventing transmission [53]. If outbreaks persist within a team, the bacterial carrier status of members can be assessed by cultivating crural areas, nasal passages can be established [51], and appropriate treatment can be instituted.

### 4.3. Pyomyositis

Pyomyositis is a purulent infection of skeletal muscle that manifests with abscess formation [54], in which the most common organism isolated is SA [55,56]. Although pyomyositis is more prevalent in tropical countries, it seems that this disorder is no longer confined to tropical regions [54], and can occur in immunocompetent patients [57,58]. Common risk factors for pyomyositis are immunodeficiency, trauma, concurrent infections and malnutrition [59,60]. Recently, it has been proposed that strenuous exercise may be a possible risk factor. Another risk factor that emerges from contact sports is muscle manipulation (physiotherapy), which can represent an additional, albeit rare, outbreak of infection [61].

### 4.4. Septic Arthritis

Septic arthritis represents a bacterial infection in a joint, and can be caused by other infectious agents, such as fungi and mycobacteria [62]. SA, including methicillin-resistant SA, infection is the most common cause of this disease. The sepsis is commonly bacterial in origin, and most often affects the joints of the lower extremity. Sepsis can result from penetrating wounds, surgery, or the hematogenous spread of bacteremia from a distant infection. Traumatic injuries to the soft tissue of a joint may increase the joint’s susceptibility to these haematogenously seeded infections. Septic arthritis is a relatively rare condition, and therefore it may not be considered initially in the differential diagnosis of joint pain in a competitive athlete [63]. Clinical suspicion should be aroused when the patient has a disproportionately painful joint and any other systemic signs of infection. Early detection and treatment make this athlete’s successful outcome and rapid return to competition possible.

### 4.5. Pubic Osteomyelitis

Osteomyelitis is an acute or chronic infection caused by bacteria. It affects one in five thousand people. SA can cause pubic osteomyelitis, which must be suspected in athletes who have pain in the hip or pubis accompanied by a feverish state. The infection that causes osteomyelitis often occurs in another part of the body, but it spreads to the bones through the blood. The bone can be predisposed to infections due to trauma or a pre-existing injury. In adults, the vertebrae and pelvis are most commonly affected [64,65]. When the bone has been infected, pus is produced inside the bone tissue, which can result in an abscess, which deprives the bone of blood. Osteomyelitis is chronic when bone tissue "dies" as a result of blood deficiency. Chronic intermittent infection can persist for years. Antibiotics will be given to destroy the bacteria causing the infection. For infections that do not heal, surgery may be needed to remove the dead bone. Antibiotics are assigned for at least 6 weeks after surgery.

### 4.6. Endocarditis

**Figure 5:**
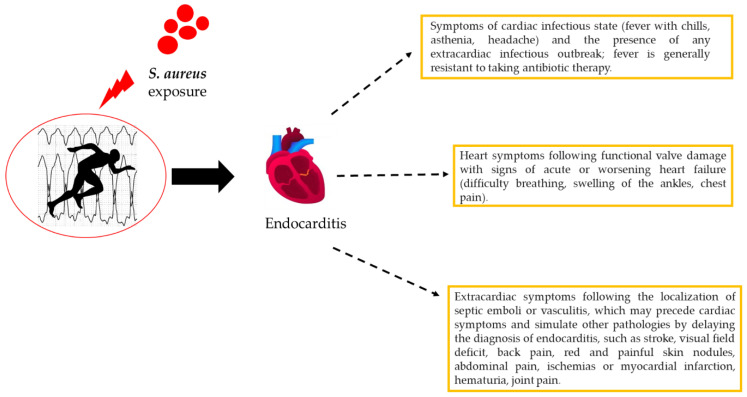
*Staphylococcus aureus* endocarditis.

Infective endocarditis (IE) is one of the most serious complications of SA infection, including methicillin-resistant SA (MRSA) [66,67,68]. In addition, those with unknown genetic cardiac disorders have a high risk of endocarditis [69,70,71,72]. Infective endocarditis is uncommon in healthy patients, but this complication can also occur in athletes (Figure 5). IE is a cardiac disorder caused by pathogenic microorganisms invading the endocardial surface, which affects intracardiac structures in contact with the blood, including large intrathoracic vessels and intracardiac devices. The epidemiology of SA endocarditis has changed in the past few decades, and this bacterium has become the leading cause of infective endocarditis in many areas of the world [73] as cases of MRSA infection have increased. SA represents the main cause of the pathogenic microorganism that causes IE in developed countries [66,74].

SA endocarditis, as well as all endocarditis, recognizes three key elements [75]: i) the anatomical substrate, ii) the trigger factor and iii) modulating factors. The underlying cause of the occurrence and development of IE is often a prosthetic heart valve replacement, acquired heart valve disease, intravenous drug abuse, congenital heart disease or cardiac implanted devices. However, the trigger factor for infective endocarditis is a transient bacteremia. SA bacteremia (SAB) is complicated by IE in 10–30% of cases, and it is associated with a poor prognosis [76,77,78]. Furthermore, the response of the immune system determines the course of the disease [75].

Community-associated MRSA (CA-MRSA) seems to threaten the young and healthy, especially individuals involved in athletic activities. Although there is a real risk of developing bacteremia in athletes because SA infection is very common, to date the cases of infective endocarditis described among athletes are limited to case reports. May et al. [79] reported the case of a 21-year-old collegiate wrestler admitted to hospital for abdominal pain. After ruling out abdominal causes with a diagnostic laparoscopy, the suspicion of endocarditis was raised. The two-dimensional echocardiogram showed a vegetative lesion on the aortic valve, and tricuspid and mitral valve insufficiency. A further echocardiographic exam highlighted a nodular thickening of the noncoronary cusp of the aortic valve. An elevated white blood cell count, a temperature of 40 °C, a blood culture positive for SA and echocardiographic findings were consistent with SA endocarditis. Although the wrestler did not show up with a full-blown skin infection, he said he struggled with open wounds. The evidence did not link the wrestler’s wounds to the contamination found on the wrestling mat; however, the authors still stressed the need for adequate hygiene and wound care among wrestlers, along with the ordinary cleaning of mats and other equipment that could represent contagion vectors.

The current guidelines of the European Society of Cardiology (ESC), published in 2015, suggest the use of Duke criteria for diagnosis, and IE is defined by the presence of two major criteria, one major and three minor criteria, or five minor criteria [66]. These are based on clinical symptoms, laboratory parameters, imaging and microbiology. 

A report of cardiac involvement in MRSA infection was recently described by Yokomori et al. [80]. A 20-year-old collegiate athlete presented life-threatening infections caused by MRSA USA300 Clone with sepsis, septic pulmonary emboli, skin and soft tissue infections with iliofemoral deep venous thrombosis, and suspected endocarditis. Other cases of infective endocarditis in athletes concern, more frequently, streptococci [81].

Beta-Lactams are the cornerstone of treatment for methicillin-susceptible SA (MSSA) endocarditis [80,81]. Regarding MRSA endocarditis, the treatment and recovery are more complex. Despite limitations concerning efficacy and toxicity, and the problem of tissue penetration, vancomycin is the gold standard for the treatment of MRSA endocarditis by the ESC guideline (IB recommendation), and daptomycin has also been recommended by IIB [66]. In the case of failure or drug toxicity, cocktails of several antibiotics are used to treat resistant infections.

For all of these reasons, in addition to the recommended hygiene guidelines, echocardiographic screening for athletes in risk sports would be useful to detect any predisposing conditions (e.g., asymptomatic congenital heart disease and valve prolapses).

## 5. *S. aureus* Identification

**Figure 6:**
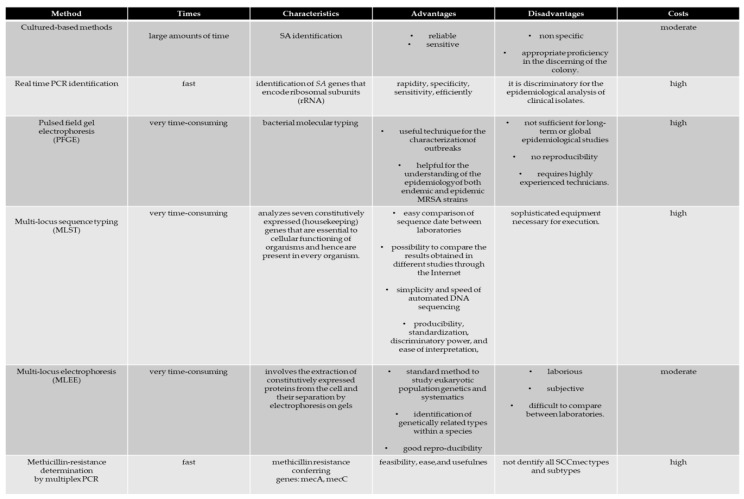
Common laboratory methods for the diagnosis of *Staphylococcus aureus*.

The birth of an outbreak of SA within a sports club (a team made up of several individuals) can certainly be counteracted by both an early diagnosis and correct information on routes that could reduce transmission, and thus have a social and economic impact which is less serious than expected [1,5]. Furthermore, when an outbreak of SA occurs, the main objective must be to isolate all of the vectors of the disease that can reside in both the players and the technical staff. Consequently, at the first hint of the birth of an SA outbreak, isolation tests of the pathogen must be performed. To date, this is the most reliable and sensitive method that can be used; the use of these tests is essential for accurate SA outbreak surveillance (Figure 6). However, it is important to note that these tests also isolate many non-specific SAs from the anterior nostrils of the nose or wound. Therefore, the lack of specificity of these methods influences the surveillance of SA infections.

Currently, both salted mannitol agar with egg yolk and Baird-Parker agar media are explicitly used for SA isolation. The preparation of these media takes a long time, and consequently the labor costs increase. In addition, this method requires a high operator competence to discriminate colonies.

In recent years, molecular typing methods have significantly improved our knowledge of SA transmission, thus representing powerful tools for tracking individual strains and detecting MRSA strains [23].

The lack of data on the incidence of SA infection among athletes has prevented active surveillance, thus leading to a failure in the prevention of infections. In the field of infection control, our understanding of SA transmission is limited by the methods used to determine the relationship between microorganisms in the context of time and space. Conventional typing methods include sequential multi-locus typing (MLST), pulsed field gel electrophoresis (PFGE) [65], spa typing [68,73] and multi-locus electrophoresis (MLEE) [73]. These diagnostic methods have contributed to a detailed description of the SA population by providing a description of the significant lineages associated with infections due to poor health and hygiene standards in different countries, and by permitting the monitoring of the appearance, dispersion and decline of the disease [73]. The application and interpretation of microbial typing tools in epidemiological studies requires the understanding of their limitations. In addition to reliability, a technique is considered valid when its capacity to discriminate between strains is satisfactory, and a biological basis for the grouping of strains with apparently distinct types is possible [82].

However, when attempting to investigate the finer details of SA outbreaks, these conventional typing methods have serious limitations [83]. Therefore, it is preferable that the typing of open reading frames (POT) be applied, which has been developed as a genotyping tool based on the multiplex polymerase chain reaction (PCR) [84]. 

Molecular techniques have been the preferred method for identifying microorganisms because of their higher specificity and sensitivity. These techniques are easier, less expensive and more rapid than past methods. In some cases, the test results are available within as little as 1 h. The PCR technique has the distinct advantages of rapidity, specificity, sensitivity, efficiency and the use of less of the sample compared to culture-based methods [85]. 

The POT methods have been applied to study nosocomial epidemics of MRSA, showing a high discriminatory power [86,87]. Although strategies using molecular genotyping have been able to successfully detect the presence of SA infection, they are expensive compared to standard culture methods [82,83,84,85,86,87,88,89,90,91,92]. In this scenario, the development of a reliable laboratory method that can be readily adopted by general diagnostics laboratories to improve and accelerate the capacity of these tests is increasingly needed.

## 6. Conclusions

**Figure 7:**
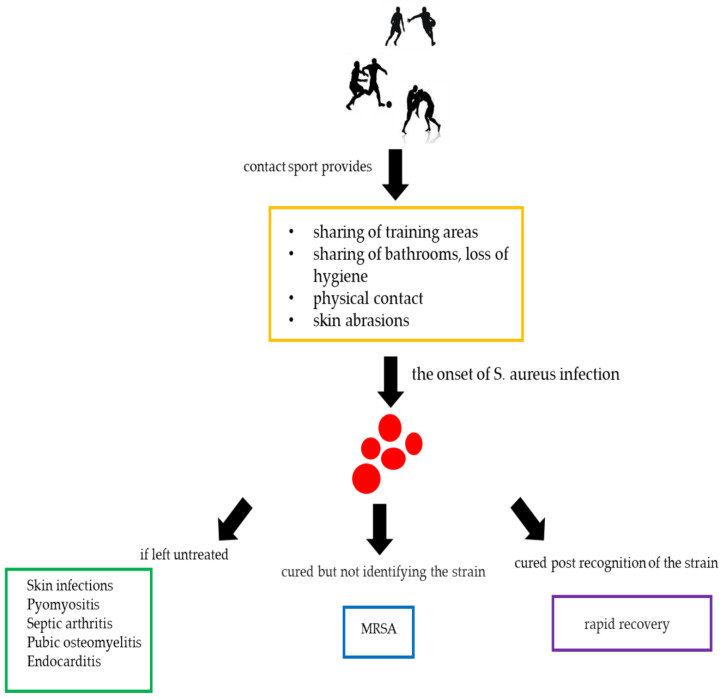
Contact sports and *Staphylococcus aureus.*

Infectious and easily transmitted diseases are the most common disorders that afflict athletes. Diagnosis and treatment are similar to those for non-athletes. However, high-level athletes are often at a higher risk due to their physiology, their lifestyles, their personal hygiene and public health practices. Furthermore, it is known that the body of an athlete is subjected to various physical “stresses”, which can represent a fertile ground for the establishment of infections, such as those caused by SA (Figure 7). Consequently, the need for faster diagnoses and more efficient therapies is increasingly evident, in order not to destabilize the athlete’s competitive activity, allowing a quick return to the game.
